# Time-Interval-Guided Event Representation for Scene Understanding

**DOI:** 10.3390/s25103186

**Published:** 2025-05-19

**Authors:** Boxuan Wang, Wenjun Yang, Kunqi Wu, Rui Yang, Jiayue Xie, Huixiang Liu

**Affiliations:** School of Automation, Beijing Information Science and Technology University, Beijing 102206, China; 2023010684@bistu.edu.cn (B.W.); 2023010747@bistu.edu.cn (W.Y.); 2023010675@bistu.edu.cn (K.W.); 2023010585@bistu.edu.cn (R.Y.); 2024010715@bistu.edu.cn (J.X.)

**Keywords:** event camera, static imaging, time interval, intensity frames

## Abstract

The recovery of scenes under extreme lighting conditions is pivotal for effective image analysis and feature detection. Traditional cameras face challenges with low dynamic range and limited spectral response in such scenarios. In this paper, we advocate for the adoption of event cameras to reconstruct static scenes, particularly those in low illumination. We introduce a new method to elucidate the phenomenon where event cameras continue to generate events even in the absence of brightness changes, highlighting the crucial role played by noise in this process. Furthermore, we substantiate that events predominantly occur in pairs and establish a correlation between the time interval of event pairs and the relative light intensity of the scene. A key contribution of our work is the proposal of an innovative method to convert sparse event streams into dense intensity frames without dependence on any active light source or motion, achieving the static imaging of event cameras. This method expands the application of event cameras in static vision fields such as HDR imaging and leads to a practical application. The feasibility of our method was demonstrated through multiple experiments.

## 1. Introduction

Recovering scenes under extreme lighting conditions presents a challenge for traditional frame-based cameras, which are often limited in capturing a broad luminance range in real-world scenarios due to their low dynamic ranges [1]. Event cameras, exemplified by the dynamic vision sensor (DVS) [2], offer a novel solution by adopting a distinct imaging mechanism. Instead of measuring the absolute light intensity of a scene to generate images, event cameras respond exclusively to changes in brightness, producing events asynchronously. In this way, event cameras offer several advantages [3], including exceptionally high temporal resolution (in the order of microseconds), a high dynamic range (up to 120 dB), low latency, and low power consumption. Since event cameras exclusively respond to changes in light intensity, they are mainly deployed in dynamic visual fields [4,5,6,7]. Researchers utilize event cameras to replace frame cameras to deal with tasks such as tracking [8,9,10,11], SLAM [12,13], and dynamic obstacle avoidance [14,15,16,17].

In addition to the progress in static scene analysis, recent efforts have focused on dynamic object pose estimation using event cameras. Liu et al. proposed a line-based method that extracts object lines directly from events and estimates poses without known 2D-3D correspondences, followed by continuous tracking via robust event-line alignment [18]. Extending this idea to aerospace applications, Liu et al. further introduced a stereo event-based pose tracking framework for uncooperative spacecraft, combining line reconstruction from stereo event streams with continuous optimization over 6-DOF motion parameters [19]. Complementing these advances, Yu et al. investigated dynamic visual scene decoding from retinal neural spikes, leveraging deep neural networks to reconstruct visual stimuli and assess decoding quality under varying noise and trial conditions, offering insights into visual neural coding and its implications for brain–machine interfaces [20]. These works collectively demonstrate the versatility of event-based sensing and neural decoding in addressing high-speed perception and cognitive reconstruction tasks. Domínguez-Morales et al. [21] designed a real-time neuromorphic stereo vision system with a novel FPGA-based calibration method inspired by human vision. Jiao et al. [22] proposed a comprehensive LiDAR and event camera calibration framework based on automatic checkerboard tracking and globally optimal optimization. Muglikar et al. [23] developed a calibration method for event cameras using neural network-based image reconstruction without requiring active illumination. Zhang [24] introduced a flexible and simple camera calibration technique using a planar pattern observed from multiple orientations.

In this paper, we propose a new method to model noise behavior and introduce a novel method for reconstructing static scenes. Specifically, we establish a correlation between the temporal information of events and the relative light intensity of the scene, facilitating precise reconstruction with the sole requirement of extracting and analyzing event timestamps. To the best of our knowledge, this study is the first to systematically elucidate the role of noise behavior in event triggering in static scenes. It is also the first to demonstrate that the high temporal resolution of event cameras, particularly timestamps, can be leveraged for the recovery of static scenes. Our contributions are summarized as follows:

1. We propose a new method termed the “noise-based event triggering mechanism”. This method provides a probabilistic perspective to elucidate the influence of noise behavior on event triggering in static scenes. It also outlines the relationship between the event generation rate and the light intensity of the scene.
2. We present the concept of “event pairs” and demonstrate that events predominantly occur in pairs. We establish the relationship between the time interval of event pairs and light intensity. Based on this observation, we propose an innovative method to convert the high temporal resolution of event signals to the relative light intensity of the static scene.
3. We developed a practical application based on our method, namely feature detection under low illumination. Our demonstrations indicate that the time-interval-based method outperforms the integration-based method in detail recovery, thereby expanding the potential applications of event cameras in static scenarios.

## 2. Related Works

This study presents a novel approach to static imaging using event cameras, with the primary objective of addressing issues such as low contrast and texture loss in recovering static scenes under low-illumination conditions. This work falls within the intersection of event-based reconstruction and HDR imaging, so we will review the latest research in the related areas.

Benefiting from the high temporal resolution and dynamic range of event cameras, researchers have attempted to leverage these advantages to tackle demanding visual tasks, potentially replacing conventional frame cameras. Their initial challenge involves addressing the incompatibility between event cameras and 2D image algorithms, thus propelling the advancement of the field of event-based reconstruction. Henri Rebecq et al. [5] introduced a recurrent network that segments the incoming event stream into sequential spatiotemporal windows of events to reconstruct high-frame-rate (HFR) videos. Lin Wang et al. [25] proposed a method based on conditional generative adversarial networks (cGANs), employing stacks of spacetime coordinates of events as input to reconstruct high-dynamic-range (HDR) images and HFR videos. Liyuan Pan et al. [26] proposed an event-based dual integral (EDI) model, which integrates regularization terms to effectively handle image blur challenges and enhance the reconstruction of high-quality videos.

In addition to video reconstruction, there are also related works that concentrate on recovering scene light intensity, akin to our research. Tsuyoshi Takatani et al. [11] introduced a technique for obtaining bispectral difference images utilizing an event camera with temporally modulated illumination, enabling 3D shape reconstruction in water. Zehao Chen et al. [27] suggested utilizing event cameras to capture intensity changes on a pure diffusion sphere and formulated an analytical expression for radiation intensity and event flow, enabling indoor lighting estimation. Richard Shaw et al. [28] devised a multi-modal end-to-end learning-based HDR imaging system, which accomplishes HDR reconstruction by combining high-quality image information from RGB with complementary high frequency and dynamic range information from events. Jin Han et al. [29] proposed a method for recovering scene radiance by analyzing the transient event frequency during the split second of a light being turned on.

In contrast to approaches that attempt to integrate events over a period [11,30] or those dependent on active light sources [27,29], our method only requires recording the output from a brief static exposure of the event camera for a few seconds to accomplish all the necessary preparations.

## 3. Preliminaries

The event camera generates events when detecting changes in light intensity but also in static scenes without apparent variations. Thomas Finateu et al. [31] pointed out that the output of the event camera includes normal events caused by changes in light intensity and some background activities. Rui Graca et al. [32] defined these activities as junction-leakage leak events and shot noise events and proposed a second-order model to elucidate the relationship between RMS granular noise voltage and photocurrent. Gao et al. further demonstrated this in [33] by establishing mathematical formulas to quantify the relationship between the event generation rate and photon absorption rate in static scenes.

While it is widely acknowledged that voltage fluctuations due to shot noise are the primary cause of event generation in static scenes by event cameras [34,35], the relationship between event generation rate and static scene intensity remains unclear.

## 4. Time Intervals of Event Pairs

### 4.1. Noise-Based Event Triggering

To understand why an event camera can produce a stable output even in the absence of any changes in light intensity, we recorded and analyzed several sets of raw data, where we found two phenomena.

**Figure 1:**
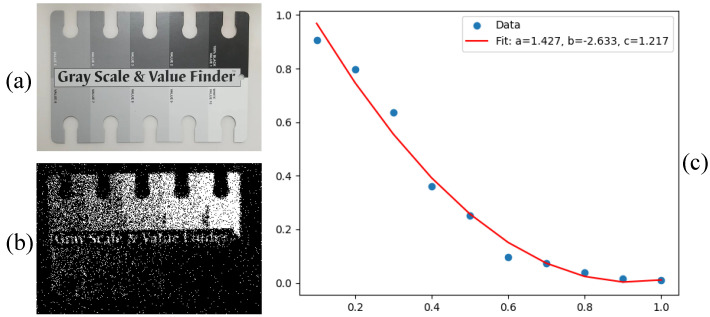
(**a**) A standard grayscale checker divides the grayscale values into ten equal parts with a 10% gradient. (**b**) Static imaging results in a single DC light source environment, reconstructed from a 5 s duration stream. There is no flickering light or changes in light intensity in the environment. (**c**) Event density vs. grayscale. We count the number of events triggered in different patches, which shows the event generation rate is strongly negatively correlated with grayscale.

**Figure 2:**
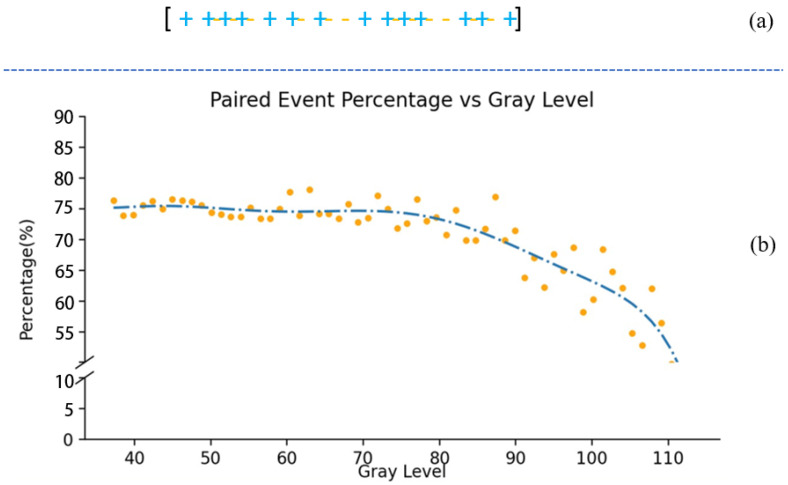
(**a**) Event pair means that only one positive event and one negative event can form an event pair, and a single event or adjacent time with the same polarity cannot form an event pair. (**b**) The relationship between the proportion of event pairs and gray values. The mean proportion reaches 73.34%, with low grayscale value pixels displaying a higher proportion than their high gray value counterparts.

1. The event generation rate in a static scene is closely associated with the scene intensity. As depicted in Figure 1, we utilize various patches within a standard grayscale checker to represent diverse illumination levels [33]. The polyfitted curve delineates the correlation between the event rate and grayscale value.
2. The majority of events in the raw stream appear in pairs, comprising one positive event coupled with one negative event, forming what we define to be an “event pair”. Figure 2 illustrates the proportion of event pairs within a set of event streams, with an average proportion of 73.34%, indicating that pairs constitute the predominant form of events.

While shot noise has been demonstrated to be the main contributor to event camera output in static scenes [32,34], the omission of consideration for other noises and random occurrences that could lead to fluctuations in current or voltage undoubtedly diminishes the robustness of the current noise theory. This is particularly evident in low illumination conditions, signifying lower photocurrent and increased susceptibility. Rahul Sarpeshkar et al. [36] have demonstrated the intrinsic unity of shot noise and thermal noise occurring in the low-power subthreshold region of the operation of an MOS transistor. It is crucial for us to employ a unified theory to explain the behavior of noise in event cameras. As the noise is filtered by the photoreceptor output stage under high light intensities, this paper concentrates on the behavior of noise under low illumination.

**Figure 3:**
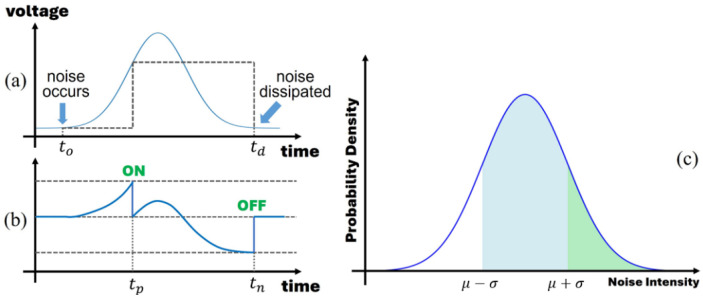
(**a**) Noise process. The occurrence and dissipation of noise can lead to fluctuations in pixel voltage, and the noise period T = td − to. (**b**) Event pair triggering. In the pre-phase and post-phase of the noise process, a positive event and a negative event are respectively triggered, with a time interval Ti = tn − tp. (**c**) Noise intensity distribution. Noise intensity follows a Gaussian distribution, and the probability of the effective noise can be expressed through the integration of the probability density function.

We assume that the noise is white and Gaussian [34,37,38], appearing constantly and randomly at each pixel. The occurrence of noise increases the pixel voltage, triggering a positive event when it crosses the ON threshold. Subsequently, the noise dissipates after reaching its intensity peak, causing the pixel voltage to decrease to a low level and triggering a negative event when it crosses the OFF threshold. We define the complete cycle of noise emergence and disappearance as the “noise process” with the duration of this cycle termed as the “noise period”, as illustrated in Figure 3a. Typical relative thresholds for event cameras range from 10% to 40% [2], indicating that as the pixel voltage increases, a higher noise intensity is needed to trigger an event. We define the minimum noise intensity required to trigger an event as the “threshold noise” and any noise surpassing this threshold can trigger events at the pixel.

As the noise intensity follows a Gaussian distribution, the probability of effective noise that can trigger events can be calculated using the following formula.

(1) $$P\left(N_{e}\right)=\int_{N_{\mathrm{th}}}^{\infty }\phi \left(x\right)\;dx$$

where $N_{\mathrm{th}}$ is the threshold noise intensity, and ϕ(x) is the probability density function of the noise intensity. Consider a scenario where there are two pixels with different values, and their threshold noises are situated at μ−σ and μ+σ, as illustrated in Figure 3c. The probability of effective noise for these two are represented by the blue and green regions in the figure, respectively. It is evident that pixels with lower threshold noise have a higher event generation rate, explaining why events are more likely to occur in low-illumination areas.

Based on the above analysis, the event generation rate is positively correlated with the Gaussian integral of the threshold noise. Considering that the noise voltage is inversely related to the photocurrent, as discussed in [32], and the photocurrent is dependent on the light intensity, the event generation rate is consequently negatively correlated with light intensity, following the Gaussian integral curve. The proof is presented in Section 4.3.

### 4.2. Intensity Reconstruction from Event Pairs

Instead of reconstructing intensities through integration, we leverage the ultra-high temporal resolution offered by event cameras, which provides the triggering time in the microsecond order. Specifically, we establish a correlation between the time intervals of event pairs and light intensity, enhancing the contrast for low-illumination reconstruction and thereby facilitating the building of a more accurate scene intensity map.

Given the close relationship between threshold noise and pixel voltage, which directly mirrors light intensity, it is theoretically feasible to reconstruct scene intensity from noise events, provided that the noise intensity is precisely measured. Accuracy in measurement is crucial during this process, emphasizing the need for a precise metric. Considering that the noise period signifies the duration of the noise process and is consequently positively correlated with noise intensity—owing to the increased time required to reach a higher peak—we utilize the time interval of event pairs that closely align with the numerical value of the noise period to characterize noise intensity. This approach is favored not only for its high accuracy but also for its accessibility, achieved simply by reading the timestamp of an event.

**Figure 4:**
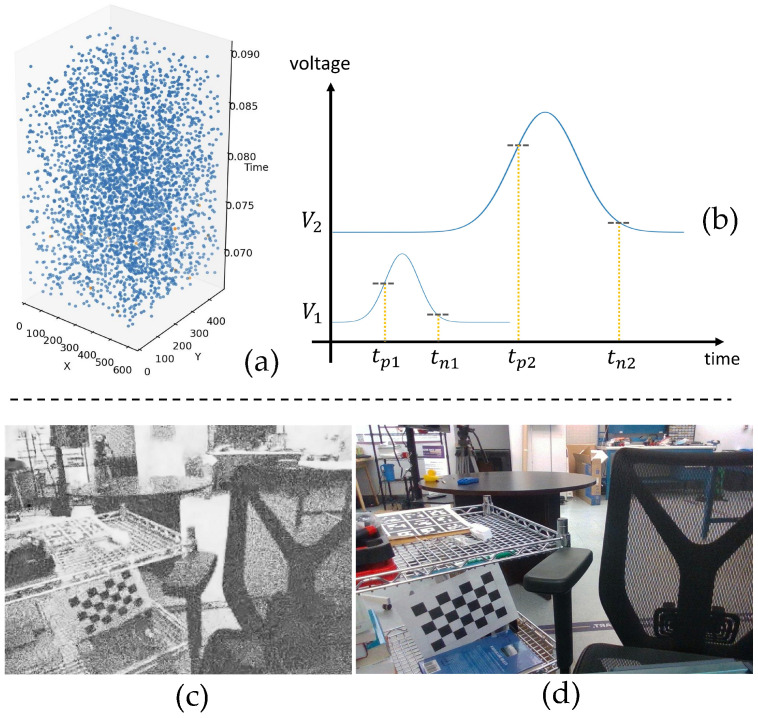
(**a**) The event stream comprises event pairs (blue) and single events (yellow). (**b**) Event pairs triggering on pixels with varying values; as the pixel voltage increases, the associated time interval lengthens. (**c**) Scene intensity map reconstructed based on time intervals, with event pairs extracted from a 5 s event stream captured by EVK1-VGA (Prophesee, Paris, France). (**d**) Ground truth obtained from the RGB camera by Intel D415 (Intel Corporation, Santa Clara, CA, USA).

Figure 4 illustrates the reconstruction of scene intensity based on the time interval of event pairs. The event stream, captured by filming a static scene for several seconds, contains event pairs in line with our theoretical description, alongside numerous single events, as depicted in Figure 4a. Consequently, it is essential to introduce a preprocessing step to enhance the ratio of event pairs. Figure 4b presents a schematic diagram of event pairs triggering. For a given pixel, the average time interval of collected event pairs during the specified duration can be calculated using the following formula:

(2) $$\bar{\left(\Delta T\right)}=\frac{1}{N}\sum_{i=1}^{N}\left(T_{i}-T_{pi}\right)$$

where *N* represents the total number of event pairs collected during the specified duration. The scene intensity can be reconstructed by normalizing the average time interval of all pixels to the (0, 255) interval, as depicted in Figure 4c.

### 4.3. Experimental Verification

In this section, we describe experiments aimed at validated our noise-based triggering method and the time-interval-based reconstruction method.

A fundamental premise of the noise-based triggering method is the assumption that the noise is white noise and conforms to a Gaussian distribution. This assumption enables us to infer that the event generation rate is correlated with light intensity, in accordance with the Gaussian integral curve. To validate this, we need to systematically alter the light intensity and record the corresponding event generation rates at different levels of illumination.

**Figure 5:**
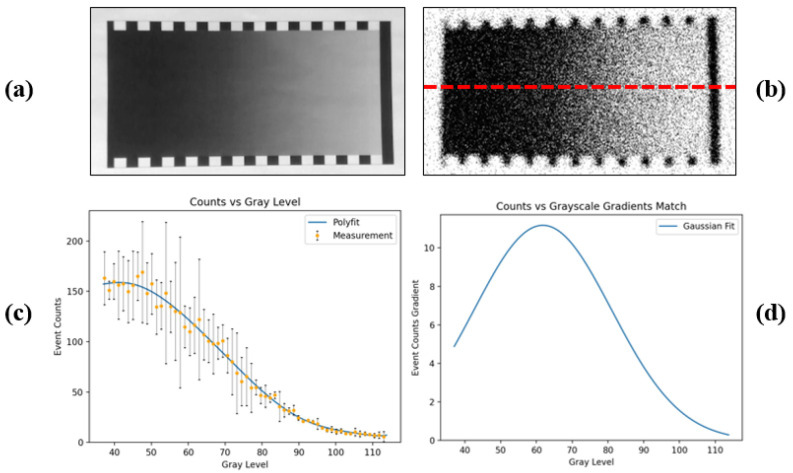
(**a**) Grayscale inspector. (**b**) Reconstructed image. (**c**) Event generation rate vs. grayscale. (**d**) The derivative curve of the event generation rate, conforming to the Gaussian distribution.

For ease of operation and quantitative analysis, we designed a grayscale checker to simulate gradual changes in light intensity, as illustrated in Figure 5a. The static imaging result is presented in Figure 5b. Using the red line as a reference, we calculated the average number of events triggered in each pixel column along the baseline and plotted how the event rate changes with grayscale to explore its relationship with light intensity, as shown in Figure 5c. The curve is obtained through polynomial fitting using the least squares method. To ascertain whether it follows a Gaussian integral, we derive its derivative curve, as illustrated in Figure 5d, where the primary body aligns with a Gaussian distribution.

**Figure 6:**
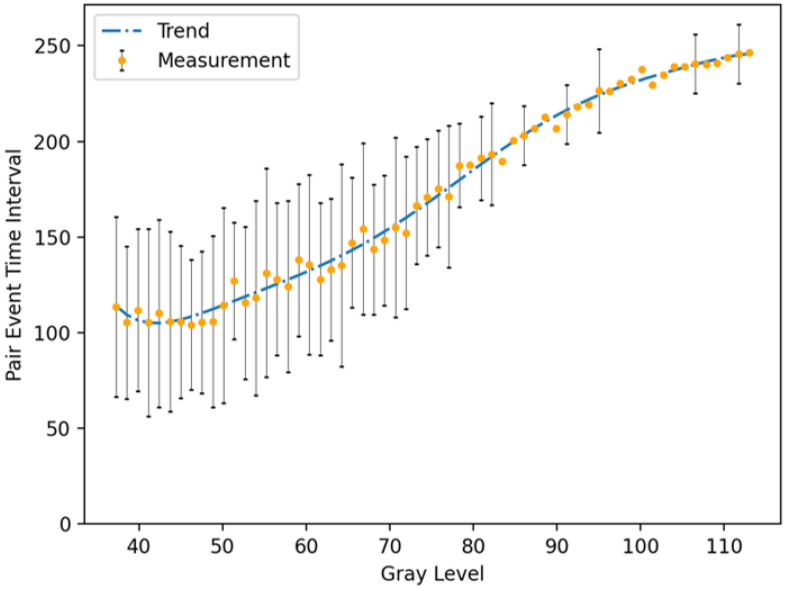
Time interval of event pairs vs. grayscale. Obtained by Prophesee EVK1-VGA.

To validate the efficacy of our time-interval-based reconstruction method, it is crucial to establish a direct mapping between the time intervals of event pairs and light intensity. We achieve this by calculating the average time interval for each pixel column along the baseline of the grayscale checker. The pixel values are obtained from the RGB camera to ensure the ground truth, and the average time intervals are measured from the pixels at the corresponding locations on the event camera. The mapping from the time interval of event pairs to grayscale is illustrated in Figure 6, revealing a robust positive correlation.

### 4.4. Time-Interval-Based Method vs. Integration-Based Method

**Figure 7:**
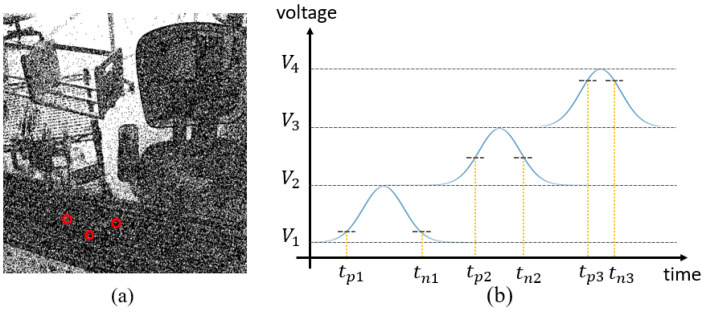
(**a**) The reconstructed image via the integration-based method exhibits low contrast, and the three selected pixels are indistinguishable. (**b**) Integration-based methods cannot extract information for differentiation, whereas time-interval-based methods can.

Let us consider a scenario where the noise of the same intensity appears on three pixels with different values, as depicted in Figure 7. Ideally, the reconstruction results for these three pixels should be distinct. However, the tiny difference in pixel voltage results in an indistinguishable triggering outcome—both a positive event and a negative event. This occurrence is frequent in low-illumination conditions, signifying low pixel voltages and consequently a slight disparity in threshold noise intensity. This subtle difference can readily induce false triggering. For the integration-based method, it merely tallies the number of triggered events and fails to extract information for differentiation. Consequently, this approach yields a low-contrast reconstruction.

**Figure 8:**
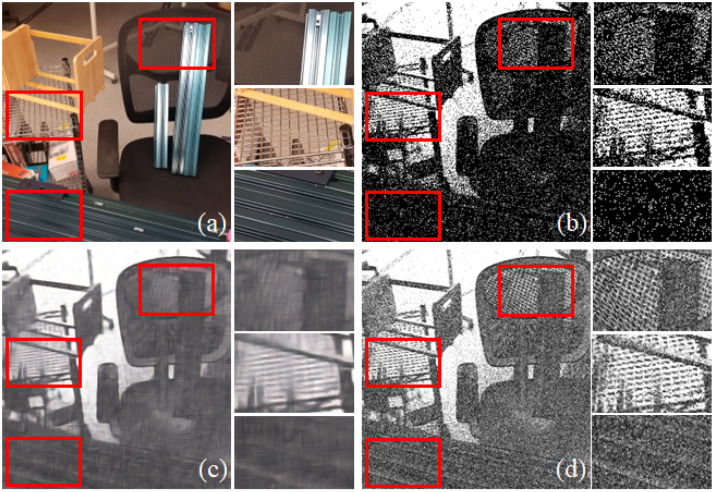
Comparison between the time-interval-based method and other approaches. (**a**) Ground truth captured by the Intel D415 camera. (**b**) Reconstruction using the integration-based method. (**c**) Reconstruction using Gao et al.’s method. (**d**) Reconstruction using our proposed method. The selected region in the frame reveals rich detail. Clearly, the time-interval-based method reconstructs scene textures more effectively, while the other methods struggle to capture such fine details.

**High Contrast.** Previous research [11,27,33] has indicated that intensity information can be reconstructed by integrating events over a period of time. However, this method encounters challenges in reconstructing intricate details, such as the texture of objects in the scene, as depicted in Figure 8b. This is attributed to the low contrast resulting from insufficient information, which can be explained by our method.

The time-interval-based method addresses this issue by leveraging the event camera’s high temporal resolution, which provides triggering times in a microsecond manner. By computing the time interval between event pairs, we achieve a high contrast that enables the distinction of pixels with low differences. This promotes a more accurate reconstruction of the scene intensity map, particularly capturing texture details under low-illumination conditions, as illustrated in Figure 8c.

To comprehensively evaluate our method, we compared the proposed approach with mainstream reconstruction techniques, including traditional integral-based methods and the method by Gao et al. [33]. The qualitative comparison results are presented in Figure 8, while the quantitative evaluation is summarized in Table 1. The quantitative analysis demonstrated that our method achieves superior PSNR scores compared to existing approaches. Furthermore, the qualitative results indicated that our reconstruction preserves finer details, such as textures, more effectively than do the competing methods.

## 5. HDR Imaging Using Event Cameras

In recent years, high-end HDR imaging technologies have advanced rapidly [39]; however, their widespread adoption remains limited due to cost and accessibility constraints.

We suggest employing event cameras for reconstructing static scenes under low illumination, addressing challenging vision tasks that conventional frame cameras struggle with due to their limited dynamic range. Two main points support this.

1. Event cameras exhibit a dynamic range exceeding 120 dB, in contrast to consumer frame cameras available at present with 40 dB. This extended range enables event cameras to capture signals under extreme lighting conditions. Theoretically, it is feasible to extract valuable information from the noisy output of event cameras.
2. In contrast to the integration-based method, our approach transforms high temporal resolution into the relative light intensity of the scene. This conversion enhances contrast, enabling us to achieve more precise reconstructions.

**Figure 9:**
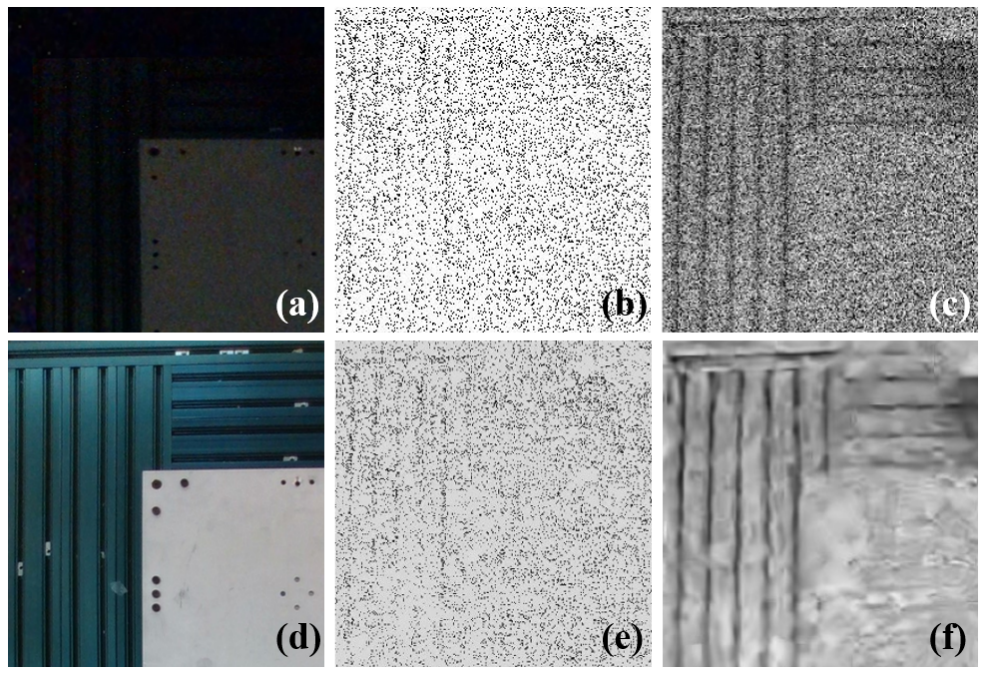
(**a**) Images captured by RGB camera under low illumination. (**b**) Images reconstructed through the integration-based method. (**c**) Images reconstructed through the time-interval-based method. (**d**) Images captured by RGB camera under normal illumination. (**e**) The denoised image of (**b**). (**f**) The denoised image of (**c**).

We effectively showcased the benefits of our time-interval-based approach through an experiment reconstructing an extremely low-light scene using an event camera. We successfully detected the texture of the table, which remained unseen by an RGB camera or by the integration-based method, as illustrated in Figure 9.

In Figure 9d, the ground truth from the RGB camera captured under normal lighting condition sis presented. Figure 9a displays an image taken by the RGB camera with a 5 s static exposure under low lighting, where the texture is challenging to discern. We reconstructed the scene from an event stream captured during a 5 s static exposures using both the integration-based method (depicted in Figure 9b) and the time-interval-based method (depicted in Figure 9c). The resulting raw frames were then denoised using the BM3D algorithm [40], as illustrated in Figure 9e,f. It can be seen that our method significantly outperformed traditional methods in reconstructing images after denoising.

**Figure 10:**
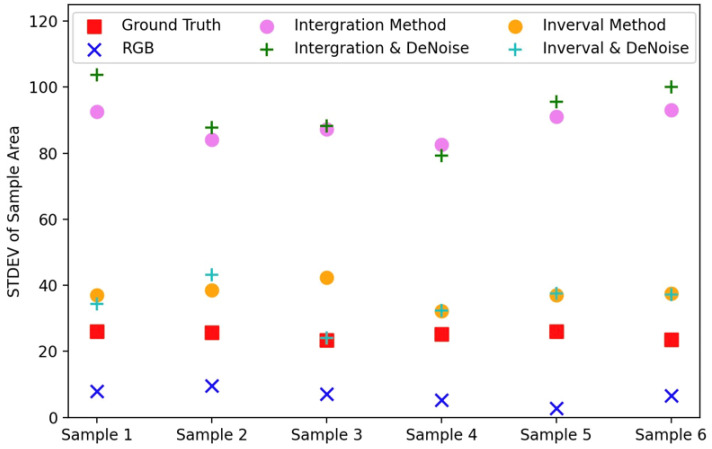
The standard deviation of pixel values within a specified pixel area was calculated for six methods, namely ground truth, the integration-based method, the time-interval-based method, RGB camera, integration-BM3D, and time-interval BM3D.

**Figure 11:**
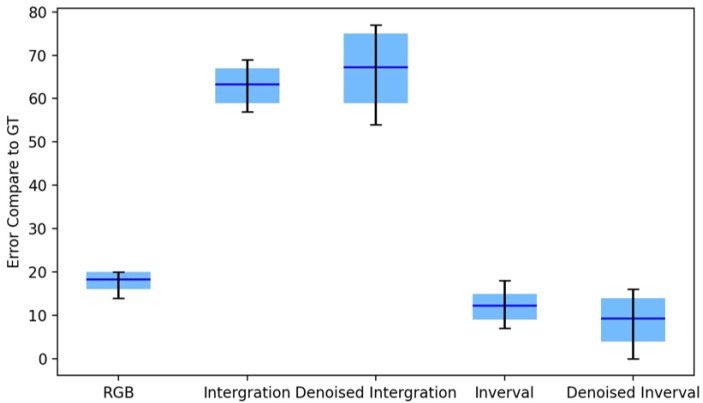
The average difference between the standard deviation of the ground truth and the standard deviation of other methods. The line y = 0 represents the ground truth.

For a quantitative assessment of the imaging capabilities under low illumination for each method (RGB camera, integration-based method, time-interval-based method),we selected the same pixel area in each image and computed the standard deviation of the pixel values within this area. The results from six independent measurements are depicted in Figure 10. The method closest to the ground truth was the time-interval-based method combined with BM3D denoising, aligning with the observations in Figure 9. Additionally, we calculated the average distance of each method from the ground truth, as presented in Figure 11. The time-interval-based method surpassed both the RGB camera and the integration-based method, highlighting the HDR imaging capability of our method under low illumination.

## 6. Conclusions

In this paper, we propose a new method to model noise behavior, elucidating its impact on event cameras for generating events in static scenes. Additionally, we introduce the concept of event pairs and establish the connection between the time interval of event pairs and the relative light intensity of the scene. Building on this theoretical foundation, we present a novel method to achieve high-contrast and accurate reconstruction of static scenes. This technology has spawned other applications, such as HDR imaging.

## Figures and Tables

**Table 1:** The quantitative comparison results between the proposed method and other approaches are presented using PSNR as the evaluation metric, with the highest values highlighted are underlined.

Method	Integration	Gao’s	Ours
PSNR	7.89	8.63	10.23

## Data Availability

The original contributions presented in this study are included in the article. Further inquiries can be directed to the corresponding author.

## References

[1] Chen X., Liu Y., Zhang Z., Qiao Y., Dong C. Hdrunet: Single image HDR reconstruction with denoising and dequantization. Proceedings of the IEEE/CVF Conference on Computer Vision and Pattern Recognition (CVPR) Workshops.

[2] Lichtsteiner P., Posch C., Delbruck T. (2008). A 128 × 128 120 dB 15 μs latency asynchronous temporal contrast vision sensor. IEEE J. Solid-State Circuits.

[3] Gallego G., Delbrück T., Orchard G., Bartolozzi C., Taba B., Censi A., Leutenegger S., Davison A.J., Conradt J., Daniilidis K. (2020). Event-based vision: A survey. IEEE Trans. Pattern Anal. Mach. Intell..

[4] Kim H., Handa A., Benosman R., Ieng S.-H., Davison A.J. (2008). Simultaneous mosaicing and tracking with an event camera. IEEE J. Solid-State Circuits.

[5] Rebecq H., Ranftl R., Koltun V., Scaramuzza D. Events-to-video: Bringing modern computer vision to event cameras. Proceedings of the IEEE/CVF Conference on Computer Vision and Pattern Recognition (CVPR).

[6] Scheerlinck C., Barnes N., Mahony R. Continuous-time intensity estimation using event cameras. Proceedings of the Asian Conference on Computer Vision (ACCV).

[7] Zou Y., Zheng Y., Takatani T., Fu Y. Learning to reconstruct high speed and high dynamic range videos from events. Proceedings of the IEEE/CVF Conference on Computer Vision and Pattern Recognition (CVPR).

[8] Barranco F., Fermuller C., Ros E. Real-time clustering and multi-target tracking using event-based sensors. Proceedings of the 2018 IEEE/RSJ International Conference on Intelligent Robots and Systems (IROS).

[9] Gehrig D., Rebecq H., Gallego G., Scaramuzza D. Asynchronous, photometric feature tracking using events and frames. Proceedings of the European Conference on Computer Vision (ECCV).

[10] Muglikar M., Gallego G., Scaramuzza D. ESL: Event-based structured light. Proceedings of the 2021 International Conference on 3D Vision (3DV).

[11] Takatani T., Ito Y., Ebisu A., Zheng Y., Aoto T. Event-based bispectral photometry using temporally modulated illumination. Proceedings of the IEEE/CVF Conference on Computer Vision and Pattern Recognition (CVPR).

[12] Mueggler E., Huber B., Scaramuzza D. Event-based, 6-DOF pose tracking for high-speed maneuvers. Proceedings of the 2014 IEEE/RSJ International Conference on Intelligent Robots and Systems (IROS).

[13] Peng X., Gao L., Wang Y., Kneip L. (2021). Globally-optimal contrast maximisation for event cameras. IEEE Trans. Pattern Anal. Mach. Intell..

[14] Falanga D., Kleber K., Scaramuzza D. (2020). Dynamic obstacle avoidance for quadrotors with event cameras. Sci. Robot..

[15] Milde M.B., Bertrand O.J.N., Benosman R., Egelhaaf M., Chicca E. Bioinspired event-driven collision avoidance algorithm based on optic flow. Proceedings of the 2015 International Conference on Event-Based Control, Communication, and Signal Processing (EBCCSP).

[16] Sanket N.J., Parameshwara C.M., Singh C.D., Kuruttukulam A.V., Fermüller C., Scaramuzza D., Aloimonos Y. EVDodgenet: Deep dynamic obstacle dodging with event cameras. Proceedings of the 2020 IEEE International Conference on Robotics and Automation (ICRA).

[17] Walters C., Hadfield S. EVReflex: Dense time-to-impact prediction for event-based obstacle avoidance. Proceedings of the 2021 IEEE/RSJ International Conference on Intelligent Robots and Systems (IROS).

[18] Liu Z., Guan B., Shang Y., Yu Q., Kneip L. (2024). Line-based 6-DoF object pose estimation and tracking with an event camera. IEEE Trans. Image Process..

[19] Liu Z., Guan B., Shang Y., Bian Y., Sun P., Yu Q. (2025). Stereo event-based, 6-DOF pose tracking for uncooperative spacecraft. IEEE Trans. Geosci. Remote Sens..

[20] Yu Z., Bu T., Zhang Y., Jia S., Huang T., Liu J.K. (2024). Robust decoding of rich dynamical visual scenes with retinal spikes. IEEE Trans. Neural Netw. Learn. Syst..

[21] Domínguez-Morales M.J., Jiménez-Fernández Á., Jiménez-Moreno G., Conde C., Cabello E., Linares-Barranco A. (2019). Bio-inspired stereo vision calibration for dynamic vision sensors. IEEE Access.

[22] Jiao J., Chen F., Wei H., Wu J., Liu M. (2023). LCE-Calib: Automatic LiDAR-Frame/Event Camera Extrinsic Calibration with a Globally Optimal Solution. IEEE/ASME Trans. Mechatron..

[23] Muglikar M., Gehrig M., Gehrig D., Scaramuzza D. How to calibrate your event camera. Proceedings of the IEEE/CVF Conference on Computer Vision and Pattern Recognition (CVPR).

[24] Zhang Z. (2000). A flexible new technique for camera calibration. IEEE Trans. Pattern Anal. Mach. Intell..

[25] Wang L., Ho Y.-S., Yoon K.-J. Event-based high dynamic range image and very high frame rate video generation using conditional generative adversarial networks. Proceedings of the IEEE/CVF Conference on Computer Vision and Pattern Recognition (CVPR).

[26] Pan L., Hartley R., Scheerlinck C., Liu M., Yu X., Dai Y. (2020). High frame rate video reconstruction based on an event camera. IEEE Trans. Pattern Anal. Mach. Intell..

[27] Chen Z., Zheng Q., Niu P., Tang H., Pan G. Indoor lighting estimation using an event camera. Proceedings of the IEEE/CVF Conference on Computer Vision and Pattern Recognition (CVPR).

[28] Shaw R., Catley-Chandar S., Leonardis A., Pérez-Pellitero E. (2022). HDR Reconstruction from Bracketed Exposures and Events. arXiv.

[29] Han J., Asano Y., Shi B., Zheng Y., Sato I. High-fidelity event-radiance recovery via transient event frequency. Proceedings of the IEEE/CVF Conference on Computer Vision and Pattern Recognition (CVPR).

[30] Galor D., Cao R., Waller L., Yates J. Leveraging noise statistics in event cameras for imaging static scenes. Proceedings of the International Conference on Computational Photography (ICCP), Spotlight Poster Demo.

[31] Finateu T., Niwa A., Matolin D., Tsuchimoto K., Mascheroni A., Reynaud E., Mostafalu P., Brady F., Chotard L., LeGoff F. 5.10 A 1280 × 720 back-illuminated stacked temporal contrast event-based vision sensor with 4.86 μm pixels. Proceedings of the 2020 IEEE International Solid-State Circuits Conference (ISSCC).

[32] Graca R., Delbruck T. (2021). Unraveling the paradox of intensity-dependent DVS pixel noise. arXiv.

[33] Gao Q., Sun X., Yu Z., Chen X. Understanding and controlling the sensitivity of event cameras in responding to static objects. Proceedings of the 2023 IEEE/ASME International Conference on Advanced Intelligent Mechatronics (AIM).

[34] Brandli C., Berner R., Yang M., Liu S.-C., Delbruck T. (2014). A 240 × 180 130 dB 3 μs latency global shutter spatiotemporal vision sensor. IEEE J. Solid-State Circuits.

[35] Indiveri G., Linares-Barranco B., Hamilton T.J., Van Schaik A., Etienne-Cummings R., Delbruck T., Liu S.-C., Dudek P., Häfliger P., Renaud S. (2011). Neuromorphic silicon neuron circuits. Front. Neurosci..

[36] Sarpeshkar R., Delbruck T., Mead C.A. (1993). White noise in MOS transistors and resistors. IEEE Circuits Devices Mag..

[37] Lichtsteiner P., Posch C., Delbruck T. A 128 × 128 120 dB 30 mW asynchronous vision sensor that responds to relative intensity change. Proceedings of the 2006 IEEE International Solid-State Circuits Conference (ISSCC).

[38] Yang M., Liu S.-C., Delbruck T. (2015). A dynamic vision sensor with 1% temporal contrast sensitivity and in-pixel asynchronous delta modulator for event encoding. IEEE J. Solid-State Circuits.

[39] Chen J., Chen N., Wang Z., Dou R., Liu J., Wu N., Liu L., Feng P., Wang G. (2025). A review of recent advances in high-dynamic-range CMOS image sensors. Chips.

[40] Dabov K., Foi A., Katkovnik V., Egiazarian K. (2007). Image denoising by sparse 3-D transform domain collaborative filtering. IEEE Trans. Image Process..

